# The Pyrazolo[3,4-*d*]pyrimidine-Based Kinase Inhibitor NVP-BHG712: Effects of Regioisomers on Tumor Growth, Perfusion, and Hypoxia in EphB4-Positive A375 Melanoma Xenografts

**DOI:** 10.3390/molecules25215115

**Published:** 2020-11-03

**Authors:** Christin Neuber, Alix Tröster, Reik Löser, Birgit Belter, Harald Schwalbe, Jens Pietzsch

**Affiliations:** 1 Institute of Radiopharmaceutical Cancer Research, Department Radiopharmaceutical and Chemical Biology, Helmholtz-Zentrum Dresden-Rossendorf, Bautzner Landstrasse 400, 01328 Dresden, Germany; c.neuber@hzdr.de (C.N.); r.loeser@hzdr.de (R.L.); b.belter@hzdr.de (B.B.); 2 Centre for Biomolecular Magnetic Resonance (BMRZ), Institute of Organic Chemistry and Chemical Biology, Johann Wolfgang Goethe-University Frankfurt a. M., Max-von-Laue-Strasse 7, 60438 Frankfurt, Germany; troester@nmr.uni-frankfurt.de (A.T.); schwalbe@nmr.uni-frankfurt.de (H.S.); 3 Faculty of Chemistry and Food Chemistry, School of Science, Technische Universität Dresden, 01069 Dresden, Germany

**Keywords:** eph receptor tyrosin kinase family, ephrins, tyrosine kinase inhibitors, regioisomers, tumor angiogenesis, tumor hypoxia, tumor perfusion

## Abstract

In a previous study, EphB4 was demonstrated to be a positive regulator of A375-melanoma growth but a negative regulator of tumor vascularization and perfusion. To distinguish between EphB4 forward and ephrinB2 reverse signaling, we used the commercially available EphB4 kinase inhibitor NVP-BHG712 (NVP), which was later identified as its regioisomer NVPiso. Since there have been reported significant differences between the inhibition profiles of NVP and NVPiso, we compared the influence of NVP and NVPiso on tumor characteristics under the same experimental conditions. Despite the different inhibitory profiles of NVP and NVPiso, the comparative study conducted here showed the same EphB4-induced effects in vivo as in the previous investigation. This confirmed the conclusion that EphB4-ephrinB2 reverse signaling is responsible for increased tumor growth as well as decreased tumor vascularization and perfusion. These results are further substantiated by microarrays showing differences between mock-transfected and EphB4-transfected (A375-EphB4) cells with respect to at least 9 angiogenesis-related proteins. Decreased expression of vascular endothelial growth factor (VEGF), angiotensin 1 (Ang-1), and protein kinase B (Akt/PKB), together with the increased expression of tissue inhibitor of metalloproteinase-1 (TIMP-1) and transforming growth factor beta-2 (TGF-β2), is consistent with the impaired vascularization of A375-EphB4 xenografts. Functional overexpression of EphB4 in A375-EphB4 cells was confirmed by activation of a variety of signaling pathways, including the Janus kinase/signal transducers and activators of transcription (JAK/STAT), rat sarcoma virus/rapidly accelerated fibrosarcoma/mitogen activated protein kinase kinase (Ras/Raf/MEK), and nuclear factor kappa-B (NFkB) pathways.

## 1. Introduction

The receptor tyrosine kinase EphB4 and its preferred membrane-bound ligand ephrinB2 play a crucial role in both physiological angiogenesis and lymphangiogenesis during embryonic development as well as in pathophysiological processes such as tumor angiogenesis [1,2,3,4,5]. Moreover, EphB4 and ephrinB2 gained increasing attention with regard to therapy resistance in different solid tumors such as head and neck squamous cell carcinoma (HNSCC), pancreatic adenocarcinoma (PDAC), and bladder urothelial carcinoma [6,7,8,9]. For example, EphB4 has been identified as a predictive biomarker for the therapy response of colorectal cancer patients receiving the vascular endothelial growth factor (VEGF)-targeting antibody bevacizumab, with an increased EphB4 expression in non-responders [6]. In line with this, combined treatment of HT-29 colon carcinoma xenografts with bevacizumab and an inhibitory EphB4-specific monoclonal antibody was superior to bevacizumab monotherapy [10]. Moreover, EphB4 overexpression was accompanied by resistance against the DNA-damaging agent cisplatin in an A375 melanoma xenograft model, also used in the present study. This effect was preventable by EphB4 inhibition, in particular, by using the EphB4 kinase inhibitor NVP-BHG712 [7]. These and other findings led to the development of Eph/ephrin-targeting agents, e.g., for therapeutic implications, which more or less specifically block the interaction of Eph receptors with their ephrin ligands and/or Eph/ephrin signaling [11,12,13]. Moreover, the Eph/ephrin system gained in importance for diagnostic (theranostic) approaches [14].

**Figure 1:**
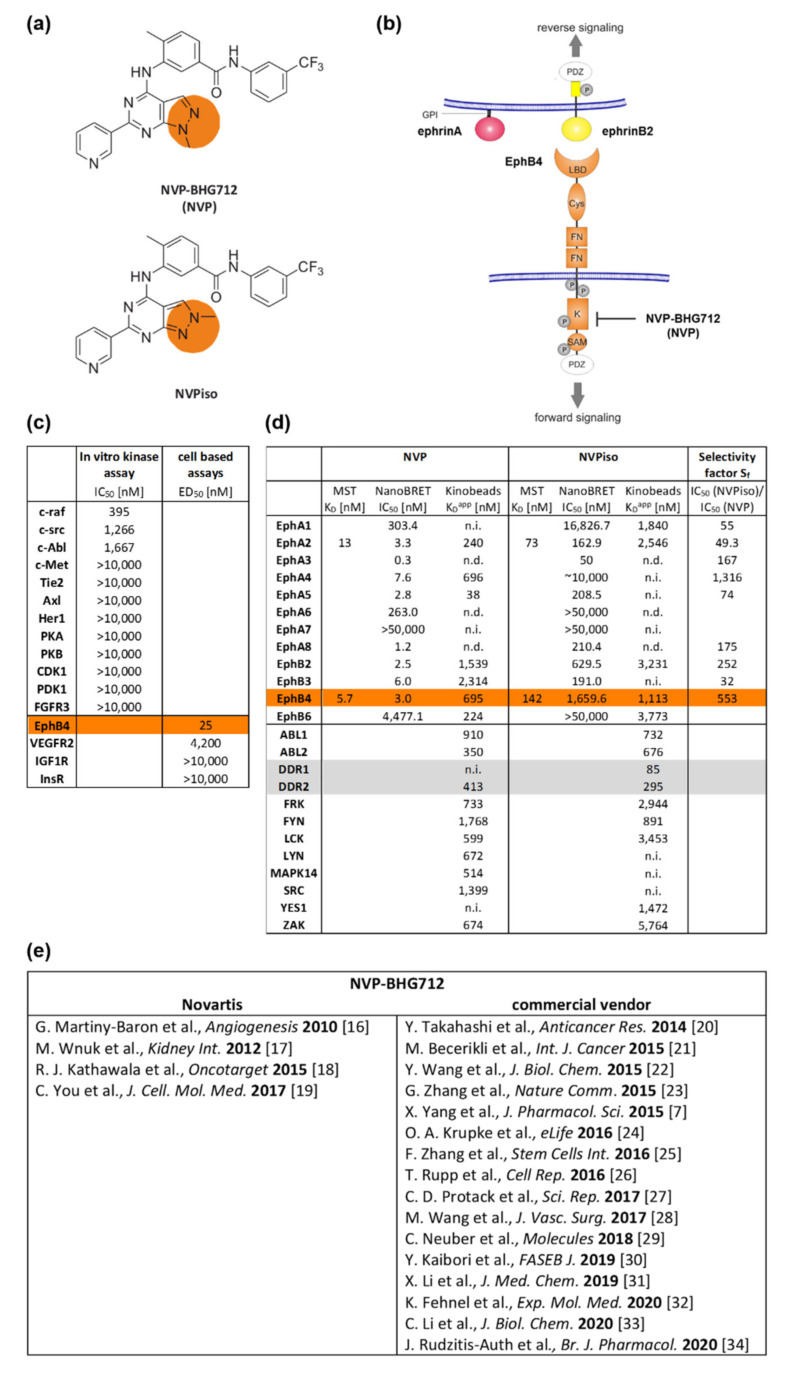
Structural and inhibitory characteristics of NVP-BHG712 (NVP) and its regioisomer NVPiso. (**a**) Structural formula of NVP and NVPiso. (**b**) Schematic structure of Eph receptors (orange) and ephrin ligands class A (red) or class B (yellow). GPI: Glycosylphosphatidylinositol anchor. PDZ: Postsynaptic density 95-disc large-Zonula occludentes-1-protein domain. LBD: Ligand-binding domain. Cys: Cysteine-rich domain. FN: Fibronectin type III domain. K: Tyrosine kinase domain. SAM: Sterile α motif. P: Potential tyrosine phosphorylation sites. Phosphorylation of EphB4 tyrosine kinase domain can be blocked by NVP-BHG712 (NVP). (**c**) Affinity data of NVP as adopted from Reference [16]. (**d**) Affinity data of NVP and NVPiso towards the Eph receptor family and off-targets, like DDR1 and DDR2, as adopted from Reference [35] (n.d., not detectable; n.i., not inhibited). (**e**) Overview of publications using NVP from Novartis or from commercial vendors.

One of the Eph/ephrin-targeting agents is the EphB4 kinase inhibitor NVP-BHG712 (NVP, Figure 1a,b), initially described in 2007 by Novartis in a patent application (WO 2007/062805) [15]. In 2010, selectivity of NVP was demonstrated in a panel of more than 40 biochemical in vitro kinase assays as well as in three further cell-based enzyme linked immunosorbent assay (ELISA) assays for phosphorylated receptor tyrosine kinases (Figure 1c) [16]. Thereby, NVP was shown to be a potent EphB4 kinase inhibitor (half maximal effective dose (ED_50,A375-EphB4_) 25 nM) with remarkably lower inhibitory potential to off-targets like EphA2, EphB2, EphB3, c-raf, c-src, and c-Abl [16]. In the recent years, a couple of studies used NVP to clarify the influence of EphB4 forward signaling, e.g., for non-cancerous and cancerous diseases (Figure 1e) [7,16,17,18,19,20,21,22,23,24,25,26,27,28,29,30,31,32,33,34]. In 2018, however, Tröster et al. demonstrated that the commercially available ‘NVP’ compound, which was purchased by six different vendors and checked for its constitution, was in fact a regioisomer of NVP (NVPiso) in each of the six samples [35]. In comparison to NVP, NVPiso is characterized by a shifted position of a single methyl group on either one of two adjacent nitrogen atoms which has implications for their inhibitory potential (Figure 1a,d, Supplementary Figures S1–S7). Whereas NVP primarily targets Eph receptor family members, the main target of NVPiso is the cancer-relevant receptor tyrosine kinase Discoidin Domain Receptor 1 (DDR1) [35]. With regard to the different Eph receptors, NVP targets the majority of Eph receptors with excellent affinities (0.3–303 nM) with the exception of EphA7 and EphB6. By contrast, NVPiso targets only half of all tested Eph receptors and these, in turn, with inferior affinities (50–630 nM) [35]. Especially with regard to the EphB4 receptor, significant differences in binding affinities of NVP and NVPiso have been observed. Whereas NVP excellently inhibits EphB4 (microscale thermophoresis (MST) assay, K_D_ 5.7 nM; bioluminescence resonance energy transfer (NanoBRET) assay, half maximal inhibitory concentration(IC_50_) 3.0 nM; Kinobeads assay, K_D_^app^ 695 nM), NVPiso showed only low inhibitory effects towards EphB4 (MST assay, K_D_ 142 nM; NanoBRET assay, IC_50_ 1660 nM; Kinobeads assay, K_D_^app^ 1113 nM), resulting in an EphB4 selectivity factor (IC_50_ NVPiso/IC_50_ NVP) of 553 [35].

In light of these findings, we decided to reevaluate our previous results [29]. Specifically, the effectiveness of the original kinase inhibitor NVP in the melanoma model was determined and compared with the data obtained with NVPiso. Within the former study, we showed that EphB4 is a positive regulator of A375 melanoma growth but a negative regulator of tumor vascularization and perfusion, which ultimately leads to increased tumor hypoxia [29]. In melanoma, tumor hypoxia is associated with a poor prognosis and unfavorable therapeutic outcome, as it contributes to metastasis as well as resistance to radiation and chemotherapy [36,37,38,39]. To distinguish between the responsibility of EphB4 forward signaling and EphB4-induced ephrinB2 reverse signaling for the observed effects, we also used ‘NVP’, at that time commercially obtained from an established vendor. Later on, the identity of the purchased ‘NVP’ was elucidated by intensive NMR-spectroscopic analysis (for further details, see the Supplementary Materials), which confirmed that it was indeed NVPiso instead of NVP. This was motivation enough to conduct a joint investigation of both groups for the direct comparison of NVP and NVPiso under the same experimental conditions, the results of which are presented herein.

## 2. Results

### 2.1. Tumor Growth

**Figure 2:**
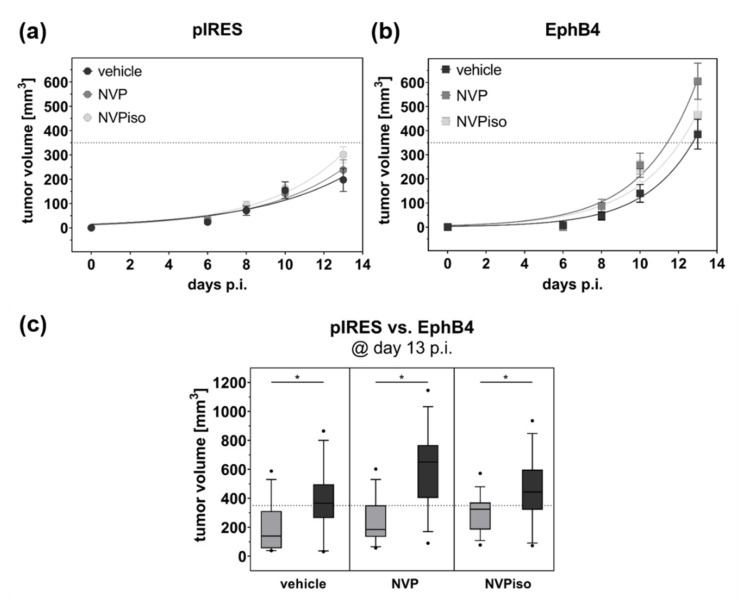
Tumor growth of A375-pIRES and A375-EphB4 tumors after treatment with vehicle, NVP, or NVPiso. Tumor volume was calculated from caliper measurement until 13 days after s.c. injection of 5 × 10^6^ A375-pIRES (**a**) and A375-EphB4 (**b**) cells. Values represent mean ± SEM (*n* = 14–15). (**c**) Comparison of calculated tumor volumes for A375-pIRES (light grey) versus A375-EphB4 (dark grey) tumors at day 13 p.i. in the three treatment groups (vehicle, NVP, NVPiso). Data within the boxes represent 10th–90th percentile (* *p* < 0.05) of ratios between A375-pIRES (light grey) and A375-EphB4 tumors (dark grey).

After subcutaneous (s.c.) injection of A375-pIRES and A375-EphB4 cells onto the left and right hind leg, respectively, of NMRI nu/nu mice, tumors were allowed to grow for about 14 days until they reached a maximum volume of 1000 mm^3^. To investigate the influence of the EphB4 kinase inhibitor NVP and its regioisomer NVPiso (Figure 1a,b) on, e.g., tumor growth, mice received 10 mg/kg body weight NVP, NVPiso, or the same volume of the used solvent (vehicle) from day 1 post tumor cell injection until the end of the experiment. As previously described by us [29], A375-EphB4 tumors grew faster than A375-pIRES tumors in the vehicle group (Figure 2a,b), resulting in higher A375-EphB4 tumor volumes at day 13 post tumor cell injection (p.i.) (Figure 2c). Neither the continuous oral administration of NVP nor that of its regioisomer NVPiso significantly affected the growth of either tumor. By trend, NVP and NVPiso slightly increased tumor growth of both A375-pIRES and A375-EphB4 tumors in comparison to the vehicle group, with only marginal differences between the two regioisomers.

### 2.2. Tumor Vascularization and Perfusion

In our previous study, A375-EphB4 tumors were characterized by a significantly decreased vascularization resulting in diminished tumor perfusion in comparison to A375-pIRES tumors [29]. Due to missing effects of blocking with the commercially acquired ‘NVP’ (from Sigma-Aldrich, later identified as NVPiso [35]), we concluded that ephrinB2 reverse rather than EphB4 forward signaling is responsible for the observed effects [29]. Therefore, in the present study, we again investigated the effects of NVPiso, this time in direct comparison to NVP, on the amount of functional tumor vasculature and tumor perfusion.

**Figure 3:**
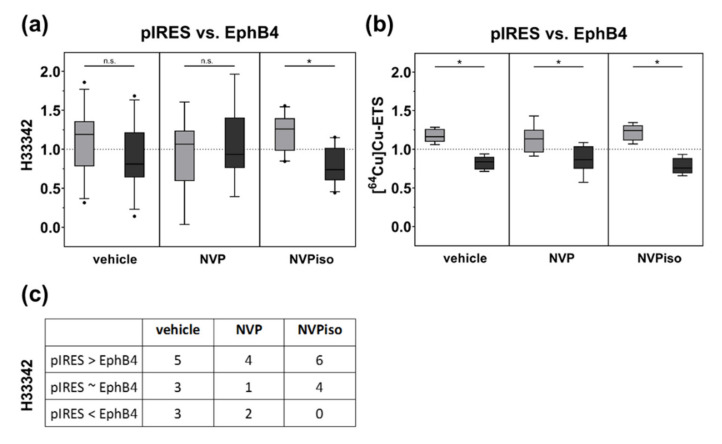
Tumor vascularization (**a**) and perfusion (**b**) of A375-pIRES and A375-EphB4 tumors after treatment with vehicle, NVP, or NVPiso. (**a**) Tumor vascularization was analyzed by i.v. injection of the fluorescence dye H33342 (*n* = 7–11) and subsequent fluorescence microscopy of 3 representative tumor sections for each tumor. (**b**) Tumor perfusion was analyzed by i.v. injection of the radiotracer [^64^Cu]Cu-ETS (*n* = 7–8) and subsequent radioluminography of 27 representative tumor sections for each tumor. Data within the boxes represent the 10th–90th percentile (* *p* < 0.05) of ratios between A375-pIRES (light grey) versus A375-EphB4 tumors (dark grey). (**c**) Data within the table represent numbers of mice with A375-pIRES tumor more, equally, or less vascularized in comparison to A375-EphB4 tumor, discriminated by a ratio cut-off value of 20%.

With regard to functional tumor vasculature, assessed by intravenous (i.v.) injection of the fluorescence dye Hoechst 33,342 (H33342) and subsequent fluorescence microscopy of tumor sections, only in the NVPiso group did we observe the already known effect, that A375-pIRES tumors are more vascularized than A375-EphB4 tumors (Figure 3a). In the vehicle group, we observed the same trend. However, this effect missed significance. By contrast, in the NVP group, we did not find significant differences in the amount of functional tumor vasculature between A375-pIRES and A375-EphB4 tumors. However, despite the missing significance of box plot data (Figure 3a), A375-pIRES tumors were more vascularized than A375-EphB4 tumors in most of the individual animals for all three groups (Figure 3c).

With regard to tumor perfusion, measured by i.v. injection of the radiotracer [^64^Cu]Cu-ETS and subsequent radioluminography of tumor sections, we observed a decrease in tumor perfusion by overexpression of EphB4 in the vehicle group (Figure 3b). This is in accordance with our previous study [29]. Moreover, we observed the same effect for the mice treated with either NVP or NVPiso. By trend, the perfusion difference between A375-pIRES and A375-EphB4 tumors was less pronounced in the NVP group, however, this could probably be caused by a higher heterogeneity of the measured tumor volumes.

### 2.3. Tumor Hypoxia

**Figure 4:**
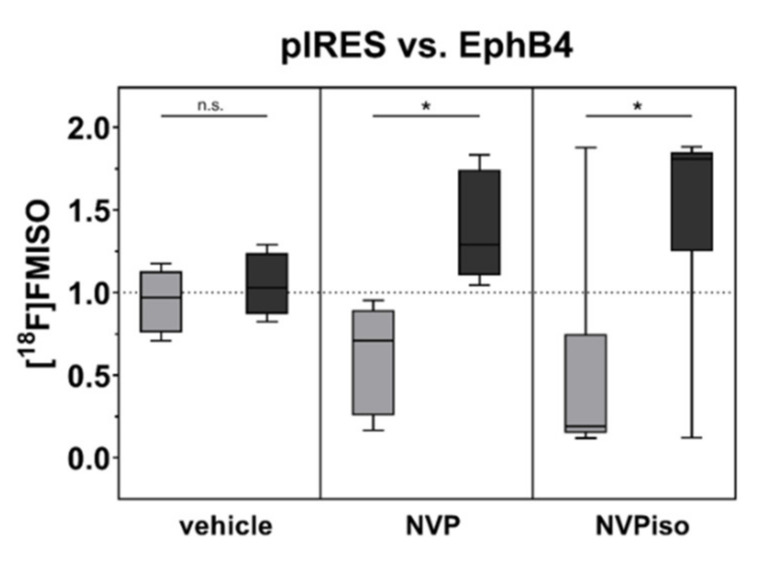
Tumor hypoxia was analyzed by i.v. injection of the radiotracer [^18^F]FMISO (*n* = 5–6) and subsequent radioluminography of 27 representative tumor sections for each tumor. Data within the boxes represent the 10th–90th percentile (* *p* < 0.05) of ratios between A375-pIRES (light grey) and A375-EphB4 tumors (dark grey).

In addition to tumor vascularization and perfusion, we compared the extent of tumor hypoxia in A375-pIRES and A375-EphB4 tumors in response to the treatment with NVP or NVPiso. As expected, diminished tumor vascularization and perfusion tend to result in an increased tumor hypoxia in A375-EphB4 tumors (Figure 4). However, this effect missed significance in the vehicle group, which is in line with our previous study [29]. Therein, further discrimination between ‘smaller’ and ‘larger’ tumors with respect to the median tumor volume sum (A375-pIRES + A375-EphB4) revealed a significantly increased tumor hypoxia in ‘smaller’ A375-EphB4 tumors [29]. However, after oral administration of NVP or NVPiso, we observed a significantly increased tumor hypoxia, determined by hypoxic fraction, in A375-EphB4 tumors without further discrimination of tumor volume. For NVPiso, this is in accordance with our previous results [29].

### 2.4. Protein Expression and Phosphorylation Array

**Figure 5:**
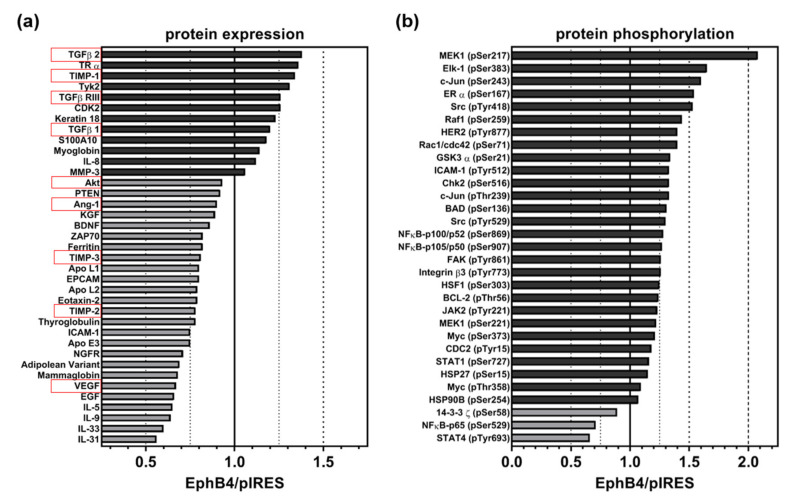
Relative change of (**a**) expression of cancer-related proteins (including angiogenesis-related proteins, highlighted in red) and (**b**) phosphorylation of signaling proteins in A375-EphB4 cells in comparison to A375-pIRES cells. Bars represent ratio of A375-EphB4/A375-pIRES cells calculated from means of average array signals from 6 independent spots for proteins with increased expression/phosphorylation (dark grey) and decreased expression/phosphorylation (light grey) in A375-EphB4 cells.

To get a first impression about the differential expression and activation of, e.g., angiogenesis-related proteins and signaling pathways in mock-transfected (A375-pIRES) versus EphB4-overexpressing A375 melanoma cells (A375-EphB4), we performed both the Cancer BioMarker Antibody Array (SCB200) and the Cancer Signaling Phospho Antibody Array (PCS248) together with the Antibody Microarray kit (KAS02) (Figure 5a,b).

In general, we observed rather weak differences in the protein expression profile of A375-pIRES and A375-EphB4 cells (Figure 5a, Supplementary Figure S8a). In total, expression of 34 out of 247 analyzed, cancer-relevant proteins differed between A375-pIRES and A375-EphB4 cells, however, all of them beneath an effect size (increase or decrease) of 50% (Figure 5a). Moreover, often one sample (A375-pIRES or A375-EphB4) was beneath the assay’s detection level (Supplementary Figure S8c). With regard to (tumor) angiogenesis-related proteins (highlighted in red), we found differences in VEGF, angiotensin 1 (Ang-1), interleukin-8 (IL-8), transforming growth factor beta-2 (TGF-β2), transforming growth factor beta receptor 3 (TGF-β R3), as well as in tissue inhibitor of metalloproteinase-1, -2, and -3 (TIMP-1, TIMP-2, and TIMP-3) expression between A375-pIRES and A375-EphB4 cells (Figure 5a, Supplementary Figure S8b). In line with the diminished vascularization of A375-EphB4 tumors, expression of the pro-angiogenic proteins VEGF and Ang-1 was decreased, whereas expression of the anti-angiogenic proteins TIMP-1 and TGF-β2 were increased in A375-EphB4 cells. However, the pro-angiogenic cytokine IL-8 and the anti-angiogenic proteins TIMP-2 and TIMP-3 behaved contrarily.

With regard to cancer-related signaling cascades, 31 out of 136 analyzed phosphorylation variants differed between A375-pIRES and A375-EphB4 cells, most of them with an effect size (increase or decrease) lower than 50% (Figure 5b). Again, often one sample (A375-pIRES or A375-EphB4) was beneath the assay detection level (Supplementary Figure S8d). Interestingly, only three proteins showed a decreased phosphorylation (14-3-3 z (pSer58), STAT4 (pTyr693), and NFkB-p65 (pSer529)) in A375-EphB4 cells. Apart from them, overexpression of EphB4 in A375 melanoma cells was accompanied by activation of a multitude of signaling pathways, e.g., the Janus kinase/signal transducers and activators of transcription (JAK/STAT), rat sarcoma virus/rapidly accelerated fibrosarcoma/mitogen activated protein kinase kinase (Ras/Raf/MEK), and nuclear factor kappa-B (NFkB) pathways. Taken together, this confirms the overexpression of functional EphB4 receptor in A375-EphB4 cells. For example, the GTPases Rac1 and Cdc42 are downstream targets of EphB receptor activation by which, in turn, Eph receptors modulate actin dynamics and cell motility [40]. Regarding angiogenesis, the decreased expression of the pro-angiogenic proteins VEGF, Ang-1, and Akt/PKB together with the increased expression of the anti-angiogenic mediators TIMP-1 and TGF-β2 (Figure 5a) is in line with the diminished vascularization of A375-EphB4 tumors.

## 3. Discussion

In our previous study, we demonstrated far-reaching consequences of EphB4 receptor overexpression in A375 melanoma cells. Using a multi-scalic and multi-modal in vivo imaging approach, we found EphB4 to be a promotor of tumor growth and hypoxia and an inhibitor of tumor vascularization and perfusion [29]. Due to bidirectionality of EphB4/ephrinB2 signaling, these effects might be initiated by either EphB4 forward signaling or ephrinB2 reverse signaling. In order to discriminate between these two possibilities, we performed a pilot blocking experiment with the small molecule EphB4 kinase inhibitor NVP-BHG712 (‘NVP’) obtained from an established vendor [29]. From the missing effects of a continuous oral administration of ‘NVP’ following the protocol published by Martiny-Baron et al. [16], we concluded in 2018 that EphB4-induced ephrinB2 reverse signaling is responsible for the observed effects on tumor growth, perfusion, and hypoxia [29]. 

In the same year, Tröster et al. found out that commercially available ‘NVP’ samples from six different vendors turned out to be in fact the regioisomer NVPiso of the EphB4 kinase inhibitor NVP-BHG712 (NVP) [35]. This regioisomer NVPiso is, in comparison to NVP, characterized by the shift of a single methyl group between two adjacent nitrogen atoms, resulting in different selectivity profiles towards the Eph receptor family and non-Eph receptor tyrosine kinases (RTKs). NVP excellently inhibits EphB4, whereas NVPiso showed only low inhibitory effects towards the EphB4 receptor [35].

The structural identity of the commercially obtained ‘NVP’ was proven to be in agreement with the 2-methyl-2*H*-pyrazolo [3,4-*d*]pyrimidine heterocycle present in NVPiso. This was judged from the ^1^H-NMR spectrum, for which the 2-methyl protons of NVPiso resonate at 4.14 ppm, while the 1-methyl protons of NVP would appear at 4.02 ppm. The chemical shift of the other protons are not significantly influenced by this constitutional isomerism [35]. ^13^C-NMR data are also in accordance with the values published for the 2*H*-pyrazolo-annelated heterocycle [35]. Furthermore, proton-carbon coupling over three bonds between the methyl protons and the carbon atom of the methine group in the pyrazole ring, as observed in the ^1^H,^13^C-HMBC spectrum at 323 K (50 °C), confirms the substitution pattern of NVPiso (see Supplementary Information). 

The present study shows that despite the significantly different inhibitory profiles of NVP and NVPiso, overexpression of EphB4 in A375 melanoma cells had similar effects on tumor characteristics independent of the treatment of the mice with vehicle, NVP, or NVPiso. This means that neither NVP nor NVPiso were able to significantly prevent the EphB4-induced effects on tumor growth, vascularization, perfusion, and hypoxia. These results support our previously published conclusion, that EphB4-mediated activation of ephrinB2 reverse signaling is responsible for the tumor growth increase as well as the decrease of tumor vascularization and perfusion, resulting in more hypoxic tumors in our A375 tumor xenograft model. This might be rather unfavorable for patients’ prognosis and therapy outcome, since tumor hypoxia substantially contributes to both chemo- and radio-resistance of tumors [29,36].

In order to better substantiate our results with this subsequent investigation, also with regard to the pathomechanisms, we analyzed both expression and activation of, e.g., angiogenesis-related proteins and signaling pathways in mock-transfected (A375-pIRES) versus EphB4-overexpressing A375 melanoma cells (A375-EphB4). 

With regard to total protein expression, we observed difference in 34 proteins, however, often only with a minor extent (effect size < 50%). Nevertheless, overexpression of EphB4 in A375 melanoma cells also influenced expression of 9 angiogenesis-related proteins (Ang-1, VEGF, Akt/PKB, TIMP-1, TIMP-2, TIMP-3, IL-8, TGF-β2, TGF-β R3). In this regard, decreased expression of the pro-angiogenic proteins VEGF, Ang-1, and Akt/PKB together with the increased expression of the anti-angiogenic mediators TIMP-1 and TGF-β2 are in accordance with the impaired vascularization of A375-EphB4 tumor xenografts. In particular, the decreased VEGF protein expression might be a result of the decreased Akt expression, and vice versa [41]. By contrast, increased expression of the pro-angiogenic cytokine IL-8 as well as decreased expression of anti-angiogenic mediators TIMP-2 and TIMP-3 seem to be contradictory. With regard to cancer-related signaling cascades, we observed an altered phosphorylation pattern for 31 phosphorylation variants as a consequence of EphB4 overexpression in A375 melanoma cells. Interestingly, only three proteins (14-3-3 z (pSer58), STAT4 (pTyr693), and NFkB-p65 (pSer529)) showed a decreased phosphorylation in A375-EphB4 cells. Apart from them, overexpression of EphB4 in A375 melanoma cells was accompanied by activation of a multitude of signaling pathways, e.g., the JAK/STAT, Ras/Raf/MEK, and NFkB pathways. Taken together, this confirms the overexpression of functional EphB4 receptor in A375-EphB4 cells. Due to heterogeneity and extensive crosstalk of (Eph/ephrin-induced) signaling cascades, it is hard to clearly assign a certain phosphorylation variant to (tumor) angiogenesis.

With regard to the particular academic motivation for this study, we can state here that the findings on NVP and NVPiso published by Tröster et al. [35] and, thankfully, critically discussed by them with ‘affected’ groups, in individual cases, do not necessarily weaken or counteract data obtained using NVPiso. The joint investigation carried out here was able to show that, in such an individual case, the results published by Neuber et al. [29] also hold up when the actual (originally intended) kinase inhibitor NVP (NVP-BHG712) was used in the same experimental model and settings.

## 4. Materials and Methods

### 4.1. Syntheses of NVP-BHG712 (NVP) and NVPiso 

NVP and NVPiso were synthesized according to the previously published procedure [35]. 

### 4.2. NMR Analysis of the Commercially Acquired ‘NVP’

Nuclear magnetic resonance spectra of commercially acquired NVP-BHG712 (Sigma-Aldrich, Taufkirchen, München, Germany; SML0333, Lot #126M4752V, referred as ‘NVP’) as used in the previously published study [29], were recorded on a 400 MR spectrometer (Agilent Technologies, Waldbronn, Germany) equipped with probe OneNMRProbe-PT at 298 or 323 K. Spectra were processed by using the program MestreNova (version 6.1.1–6384) [42]. NMR chemical shifts were referenced to the residual solvent resonances relative to tetramethylsilane. Assignments are based on the work of Tröster et al. with applying the identical atom numbering scheme (see Supplementary Materials) [35]. NMR spectra (^1^H, ^13^C, HSQC, HMBC) are shown in Supplementary Materials. 

^1^H-NMR (400 MHz, DMSO-*d*_6_, 298 K) δ (ppm) = 10.48 (br s, 1H, NH), 10.03 (br s, 1H, NH), 9.35 (br d, ^4^*J*_H,H_ = 1.5 Hz, 1H, H-19), 8.58 (dd, ^3^*J*_H,H_ = 4.8, ^4^*J*_H,H_ = 1.7 Hz, 1H, H-20), 8.53 (dt, ^3^*J*_H,H_ = 8.0, ^4^*J*_H,H_ = 1.9 Hz, 1H, H-22), 8.25 (br d, 1H, ^4^*J*_H,H_ = 1.3 Hz, H-2), 8.23 (br s, 1H, H-12), 8.07 (br d, ^3^*J*_H,H_ = 8.6 Hz, 1H, H-8), 7.92 (dd, ^3^*J*_H,H_ = 7.8, ^4^*J*_H,H_ = 1.3 Hz, 1H, H-6), 7.63–7.54 (m, 2H, H-5, H-9), 7.47–7.38 (m, 2H, H-10, H-21), 4.14 (s, 3H, N-CH_3_), 2.35 (s, 3H, CH_3_), H-15 not visible at this temperature (in agreement with Reference [35]).

^1^H-NMR (400 MHz, DMSO-*d*_6,_ 323 K) δ (ppm) = 10.19 (s, 2H, 2 × NH), 9.36 (br d, ^4^*J*_H,H_ = 2.1 Hz, 1H, H-19), 8.57 (dd, ^3^*J*_H,H_ = 4.8, ^4^*J*_H,H_ = 1.7 Hz, 1H, H-20), 8.54 (dt, ^3^*J*_H,H_ = 8.0, ^4^*J*_H,H_ *=* 2.0 Hz, 1H, H-22), 8.30–8.14 (m, 3H, H-2, H-12, H-15), 8.10 – 8.04 (m, 1H, H-8), 7.91 (dd, ^3^*J*_H,H_ = 7.9, ^4^*J*_H,H_ *=* 1.9 Hz, 1H, H-6), 7.59 (t, ^3^*J*_H,H_ = 8.0 Hz, 1H, H-9), 7.54 (d, ^3^*J*_H,H_ = 8.0 Hz, 1H, H-5), 7.47–7.36 (m, 2H, H-10, H-21), 4.13 (s, 3H, N-CH_3_), 2.36 (s, 3H, CH_3_).

^13^C-NMR (101 MHz, DMSO-*d*_6_, 298 K) δ (ppm) = 165.3 (C=O), 161.0 (C-16), 158.5 (C-17), 156.5 (C-13), 150.4 (C-20), 149.1 (C-19), 140.1 (C-7), 138.2 (C-4), 134.9 (C-22), 134.2 (C-18), 132.4 (C-1), 130.6 (C-5), 129.9 (C-9), 129.3 (q, ^2^*J*_C,F_ = 31.4 Hz, C-11), 125.8 (C-2), 125.5 (C-6 and C-15), 124.2 (q, ^1^*J*_C,F_ = 270.6 Hz, CF_3_), 123.9 (C-8), 123.3 (C-21), 119.8 (m, C-10), 116.4 (q, ^3^*J*_C,F_ = 4.0 Hz, C-12), 101.1 (C-14), 40.4 (N-CH_3_), 18.2 (CH_3_). Signal for C-3 was not detected.

^13^C (DEPT) NMR (101 MHz, DMSO-*d_6_*, 323 K) δ (ppm) = 149.99 (C-20), 148.74 (C-19), 134.41 (C-22), 130.24 (C-5), 129.33 (C-9), 125.66 (C-2), 124.98 (C-6 and C-15), 123.48 (C-8), 122.79 (C-21), 119.45 (m, C-10), 116.12 (q, ^3^*J*_C,F_ = 4.1 Hz, C-12), 39.99 (N-CH_3_), 17.64 (CH_3_).

### 4.3. Generation of A375 Melanoma Xenografts

All animal experiments were carried out according to the guidelines of the German Regulations for Animal Welfare. The protocols were approved by the local Ethical Committee for Animal Experiments (AZ DD24.1-5131/449/49). Generation of tumor xenografts was performed as described elsewhere [43]. In brief, Rj:NMRI-Foxn1 nu/nu mice were s.c. injected with 5 × 10^6^ A375-pIRES and A375-EphB4 cells, each in 100 μL 0.9% *v/v* NaCl, into the left and right hind leg, respectively. Tumor size was monitored thrice a week by caliper measurements and tumor volume was calculated using the formula V = π/6 × (tumor length × tumor width^2^). Tumor-bearing mice were included into the experiments about 14 days post tumor cell injection, when tumors reached a volume of at most 1000 mm^3^.

### 4.4. Blocking Experiments with the EphB4 Kinase Inhibitor NVP-BHG712 and NVPiso 

In order to investigate the influence of the EphB4 kinase inhibitor NVP-BHG712 (referred to as NVP) and its regioisomer NVPiso on tumor growth, perfusion, and hypoxia of A375 melanoma xenografts, mice received 10 mg/kg body weight NVP, NVPiso, or the same volume of the used solvent (10% *v/v* 1-Methyl-2-pyrrolidone (NMP), 90% *v/v* PEG300; 6.7 mL/kg body weight) by oral administration from day 1 post tumor cell injection until the end of the experiment (once a day, weekdays only), as described previously [16,43].

### 4.5. Investigation of Tumor Perfusion and Tumor Hypoxia Using [^64^Cu]Cu-ETS and [^18^F]FMISO

To assess the functional parameters of tumor perfusion and tumor hypoxia, we used the radiotracers Ethylglyoxal-bis(thiosemicarbazonato)[^64^Cu]copper(II) ([^64^Cu]Cu-ETS) and 1-(2-Nitro-imidazolyl)-3-[^18^F]fluoro-2-propanol ([^18^F]FMISO) respectively, as previously described [43]. In brief, about 15 MBq [^64^Cu]Cu-ETS (*n* = 7–8 each treatment group) or 25 MBq [^18^F]FMISO (*n* = 5–6 each treatment group) were i.v. injected into a lateral tail vein of the mouse and mice were sacrificed at 1 h p.i. ([^64^Cu]Cu-ETS) or 4 h p.i. ([^18^F]FMISO). Afterwards, tumors were resected and frozen with −20 °C cold 2-methylbutane (Sigma Aldrich, Taufkirchen, München, Germany) for cryosectioning.

### 4.6. Investigation of Tumor Vascularization Using H33342 

To assess the amount of functional tumor vasculature, mice received the fluorescence dye bisBenzimide Hoechst 33,342 trihydrochloride (H33342) as previously described [43]. In brief, mice were i.v. injected with 30 mg/kg body weight H33342 (*n* = 9–11 each treatment group) exactly 1 min before sacrification. Afterwards, tumors were resected and frozen with −20 °C cold 2-methylbutane (Sigma Aldrich) for cryosectioning.

### 4.7. Cryosectioning and Quantitative Analysis of Radioluminography ([^64^Cu]Cu-ETS, [^18^F]FMISO) as Well as Fluorescence Microscopy (H33342)

Representative tumor cryosections (5 µm, 9 consecutive tumor sections in 3 tissue depths for each tumor) were prepared using the cryomicrotom CM1850 (Leica, Wetzlar, Germany), mounted onto SuperFrostPlus object slides (Thermo Fischer Scientific, Waltham, MA, USA), and dried. Afterwards, mounted tumor cryosections were used immediately for radioluminography and, later on, for H33342 fluorescence microscopy as well as subsequent hematoxylin and eosin (H & E) staining.

Radioluminography and fluorescence microscopy images were analyzed using ROVER (region of interest visualization, evaluation and image verification) software (ABX advanced biochemical compounds, Radeberg, Germany) and Fiji respectively, as previously described [43,44]. In brief, for [^18^F]FMISO and H33342, the percentage of positive stained area within the tumor area was quantified with exclusion of image artefacts and adjacent mouse tissue indicated by H & E staining. In case of [^64^Cu]Cu-ETS radioluminography, mean intensity within the tumor sections was quantified in order to fulfill perfusion intensity rather than perfused tumor fraction. Results are illustrated as ratio between results for A375-pIRES or A375-EphB4 tumors and the mean of both in the same mouse, e.g., ratio of [^18^F]FMISO-positive hypoxic fraction (HF) for A375-pIRES = 2 × HF_pIRES_/(HF_pIRES_ + HF_EphB4_).

### 4.8. Protein Expression and Phosphorylation Array

To get a first impression about the differential expression and activation of, e.g., angiogenesis-related signaling pathways in mock-transfected (A375-pIRES) versus EphB4-overexpressing A375 melanoma cells (A375-EphB4), Cancer BioMarker Antibody Array (Full Moon BioSystems, Sunnyvale, CA, USA; #SCB200) and Cancer Signaling Phospho Antibody Array (Full Moon BioSystems, #PCS248) respectively, were performed after cell sample preparation using the Antibody Microarray kit (Full Moon BioSystems, #KAS02) according to the manufacturer’s instructions. In brief, subconfluent cell culture flasks were washed, detached, and lysed. Cleared supernatant was transferred to a gel-containing column for protein extraction by centrifugation. Protein concentration of the lysate was measured by UV absorbance spectroscopy and protein sample was biotinylated. After blocking, array slides were incubated with the biotinylated protein samples and, afterwards, with Alexa546-streptavidin (1 mg/mL). Scanning of the slides as well as image analysis has been performed by the manufacturer using a microarray scanner. In total, 247 cancer-relevant proteins and 136 cancer-relevant phosphorylation variants were analyzed in comparison to their total protein expression. Results are illustrated as relative change in expression or phosphorylation in A375-EphB4 cells in comparison to A375-pIRES cells (A375-EphB4/A375-pIRES) calculated from means of average array signals from 6 independent spots of each tumor cell sample. 

### 4.9. Statistical Analysis

All ratios between results for A375-pIRES or A375-EphB4 tumors are presented as box plots with the 10th–90th percentile. Results were tested for their statistical significance using analysis of variance (ANOVA) followed by Bonferroni’s post hoc test with significance levels set at *p*-value < 0.05 (* *p* < 0.05) using the software OriginPro 2017G (OriginLab, Northampton, MA, USA). 

## Supplementary Materials

The following are available online, Figure S1: Structure of NVPiso, Figure S2: ^1^H-NMR spectrum of commercially acquired ‘NVP’ at 298 K, Figure S3: Temperature dependence of ^1^H-NMR spectra of commercially acquired ‘NVP’, Figure S4: ^13^C-NMR spectrum of commercially acquired ‘NVP’, Figure S5: ^13^C (DEPT) NMR spectrum at 323 K of commercially acquired ‘NVP’, Figure S6: ^1^H,^13^C-HSQC spectrum of commercially acquired ‘NVP’ at 323 K, Figure S7: ^1^H,^13^C-HMBC spectrum of commercially acquired ‘NVP’ at 323 K, Figure S8: Differential expression and phosphorylation of cancer-related proteins in A375-EphB4 cells in comparison to A375-pIRES cells.
