# Supplementary material for: The Pyrazolo[3,4-d]pyrimidine-Based Kinase Inhibitor NVP-BHG712: Effects of Regioisomers on Tumor Growth, Perfusion, and Hypoxia in EphB4-Positive A375 Melanoma Xenografts
Source: Molecules. 2020 Nov 3;25(21):5115. doi: 10.3390/molecules25215115 (PMC7662635; doi:10.3390/molecules25215115)
Supplement: Supplementary file 1 [file molecules-25-05115-s001.pdf]

Supporting Information

The pyrazolo[3,4-*d*]pyrimidine-based kinase inhibitor NVP-BHG712: Effects of regioisomers on tumor growth, perfusion, and hypoxia in EphB4-positive A375 melanoma xenografts

Christin Neuber [1], Alix Tröster [2], Reik Löser [1,3], Birgit Belter[1], Harald Schwalbe [2,#], and Jens Pietzsch [1,3,*,#]

[1] Helmholtz-Zentrum Dresden-Rossendorf, Institute of Radiopharmaceutical Cancer Research, Department Radiopharmaceutical and Chemical Biology, Bautzner Landstrasse 400, 01328 Dresden; c.neuber@hzdr.de (C.N.); r.loeser@hzdr.de (R.L.); b.belter@hzdr.de (B.B.); j.pietzsch@hzdr.de (J.P.)

[2] Institute of Organic Chemistry and Chemical Biology, Centre for Biomolecular Magnetic Resonance (BMRZ), Johann Wolfgang Goethe-University Frankfurt a. M., Max-von-Laue-Strasse 7, 60438 Frankfurt am Main; troester@nmr.uni-frankfurt.de (A.T.); schwalbe@nmr.uni-frankfurt.de (H.S.)

[3] Technische Universität Dresden, School of Science, Faculty of Chemistry and Food Chemistry, 01069 Dresden, Germany

* Correspondence: j.pietzsch@hzdr.de; Tel.: +49-351-260-2622

[#] H.S. and J.P. share senior authorship.

Received: date; Accepted: date; Published: date

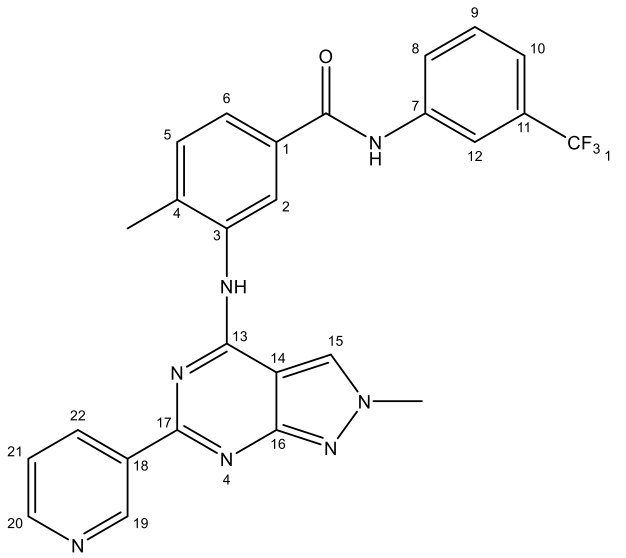

Figure S1. Structure of NVPiso. Arbitrary atom numbering as suggested by Tröster et al. [1].

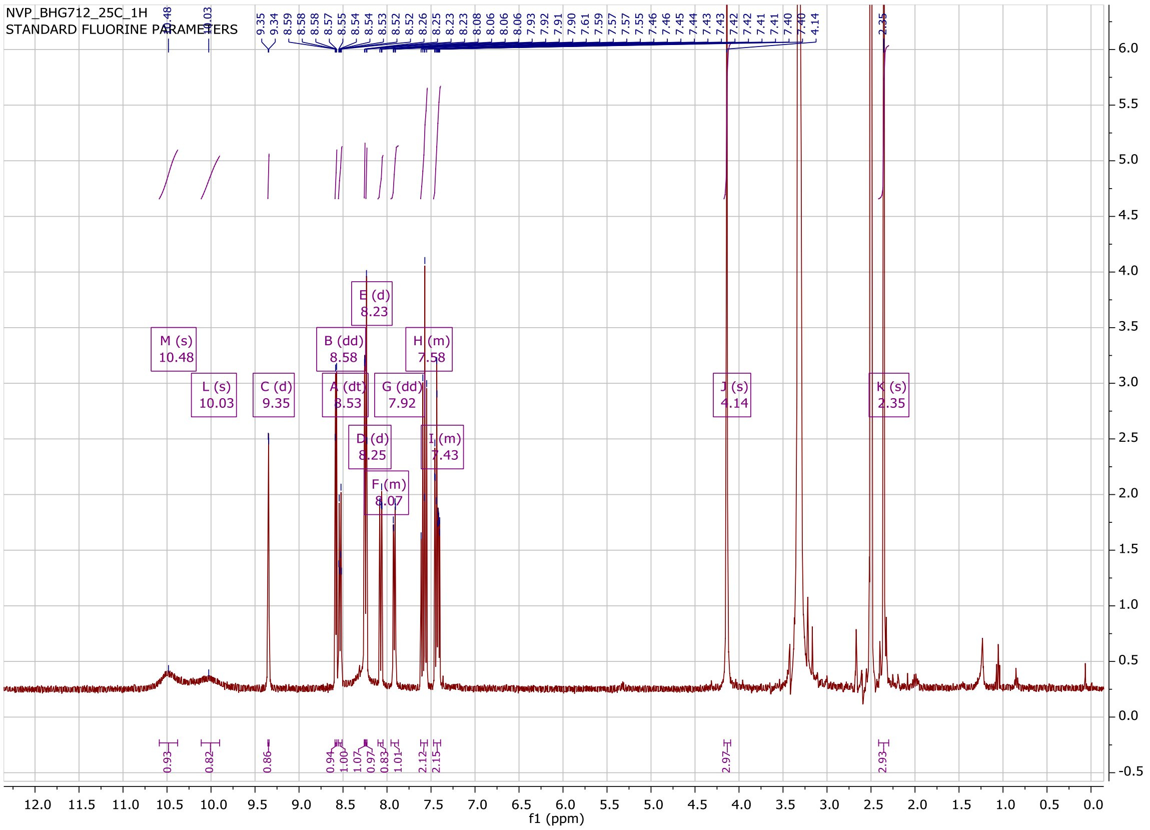

Figure S2. ^{1}H NMR spectrum of commercially acquired 'NVP' at 298 K.

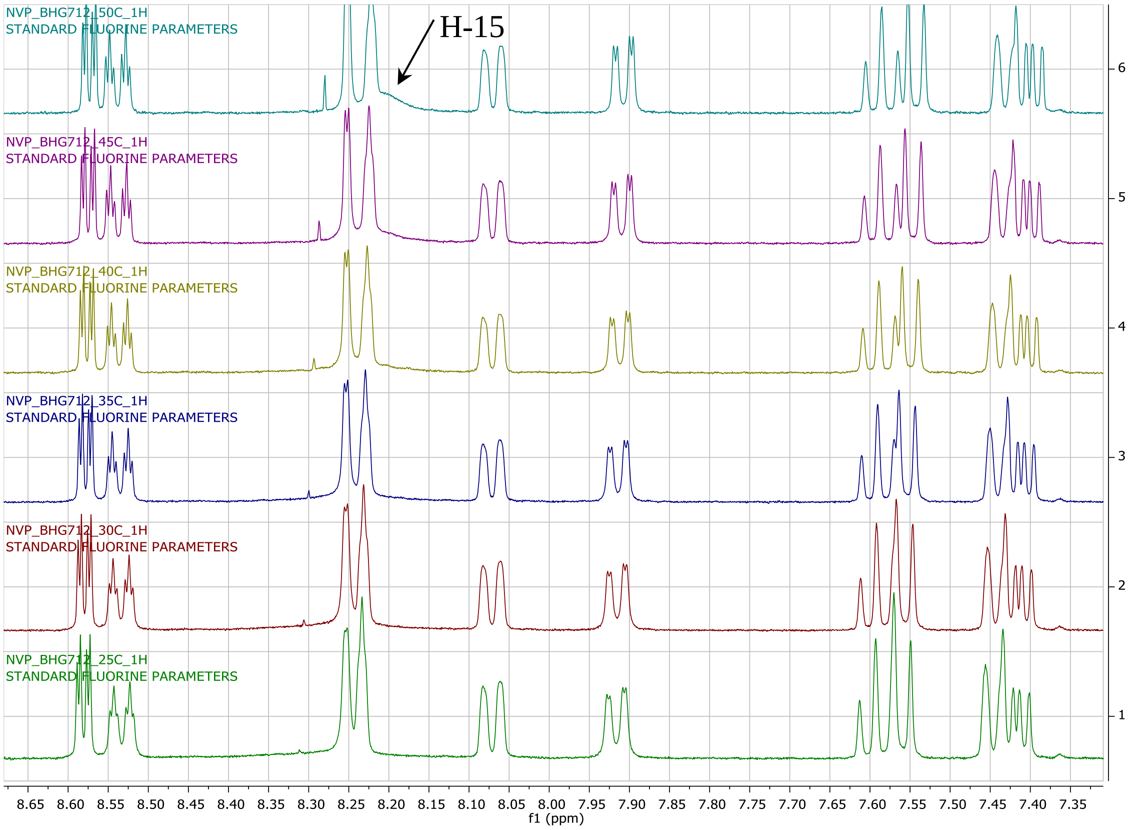

Figure S3. Temperature dependence of ^{1}H NMR spectra of commercially acquired 'NVP'. Shown is the aromatic region (signal of H-19 lies out) at 298, 303, 308, 313, 318 and 323 K to demonstrate that H-15 at

the pyrazole ring is only visible as broad singlet at temperatures above 318 K as reported by Tröster et al. [1].

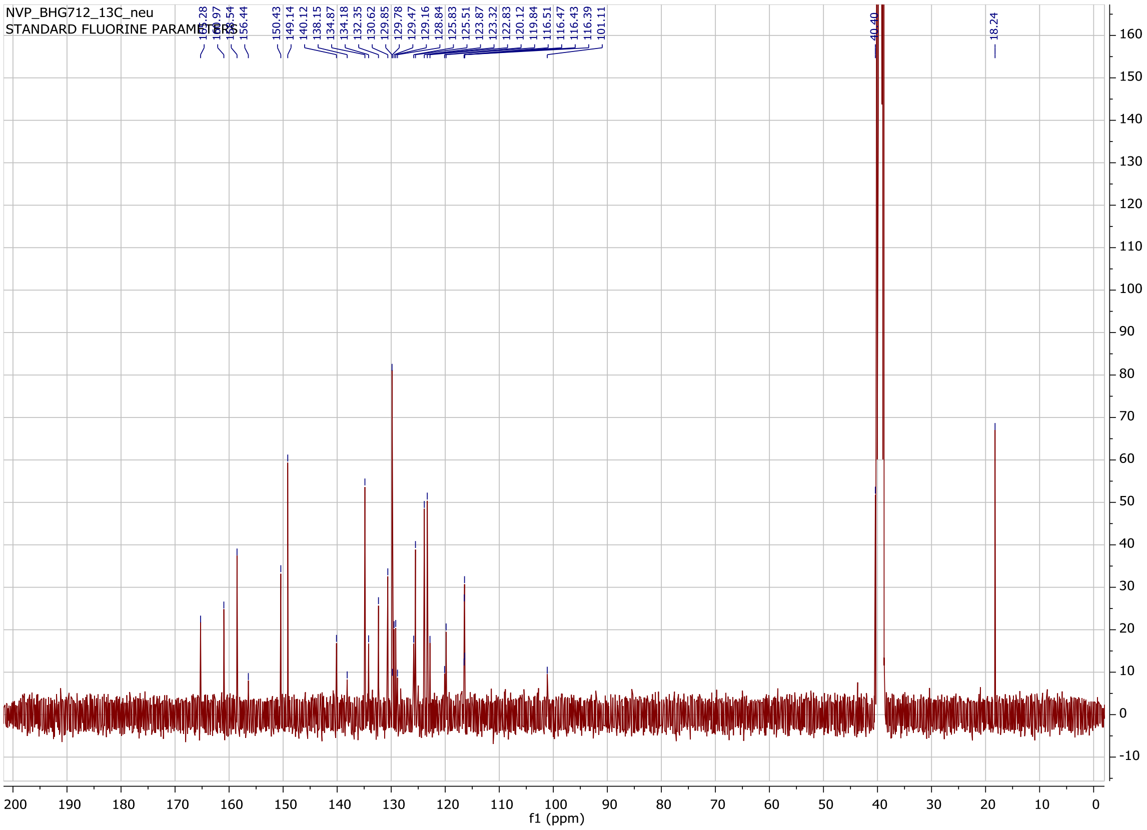

Figure S4. ^{13}C NMR spectrum of commercially acquired 'NVP' at 298 K.

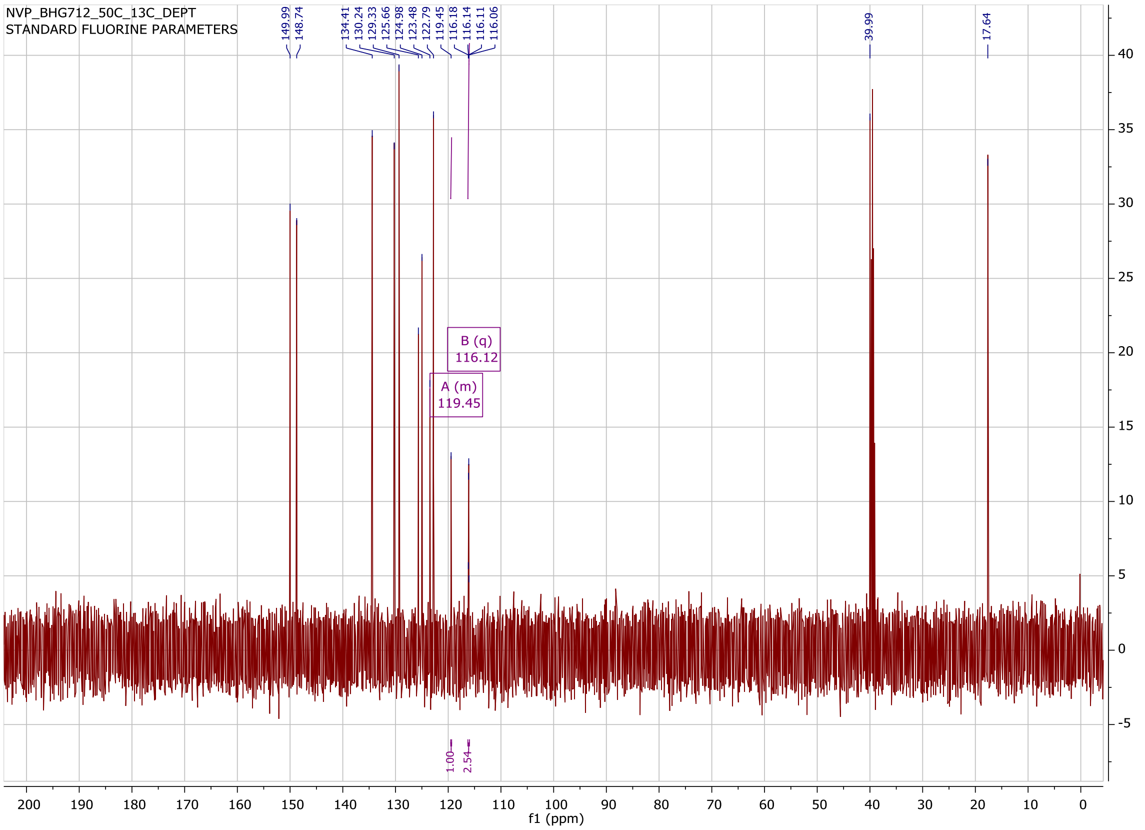

Figure S5. ^{13}C (DEPT) NMR spectrum at 323 K of commercially acquired 'NVP'.

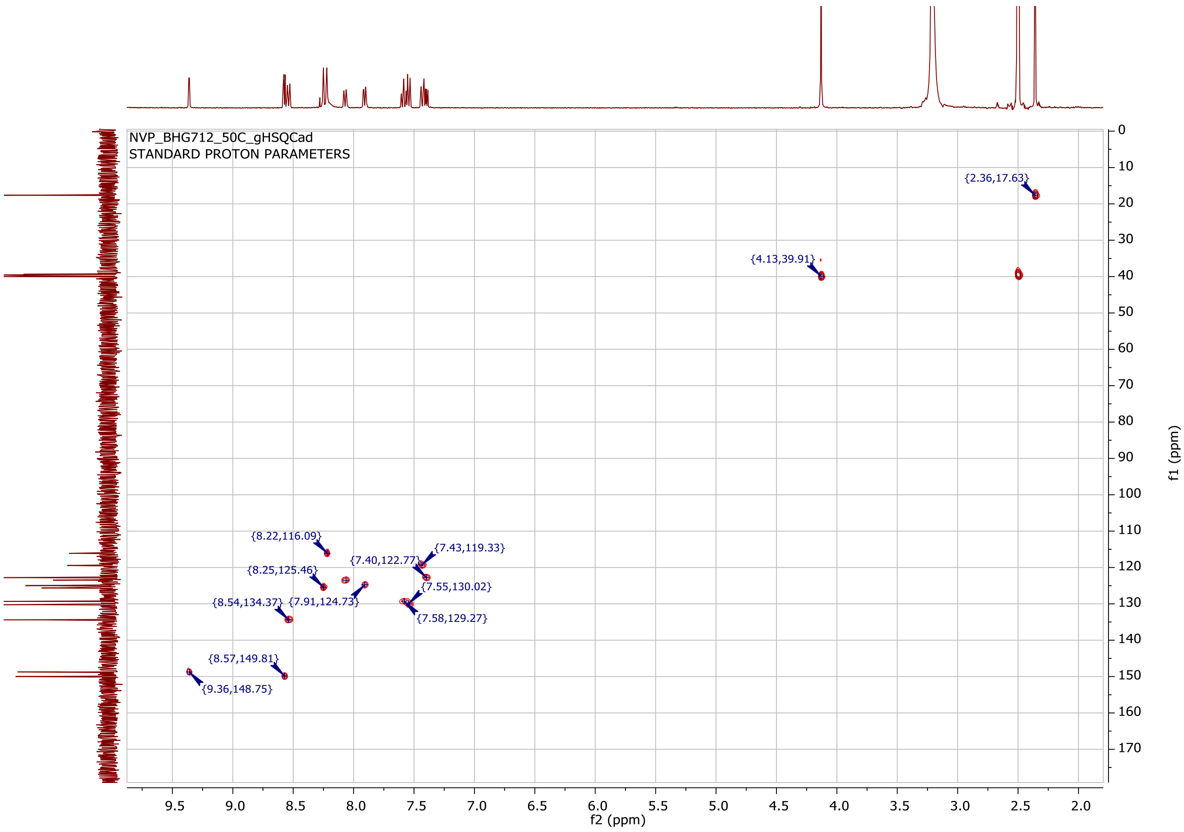

Figure S6. $^{1}H,^{13}C$ HSQC spectrum of commercially acquired 'NVP' at 323 K. The DEPT spectrum is shown on the ^{13}C edge.

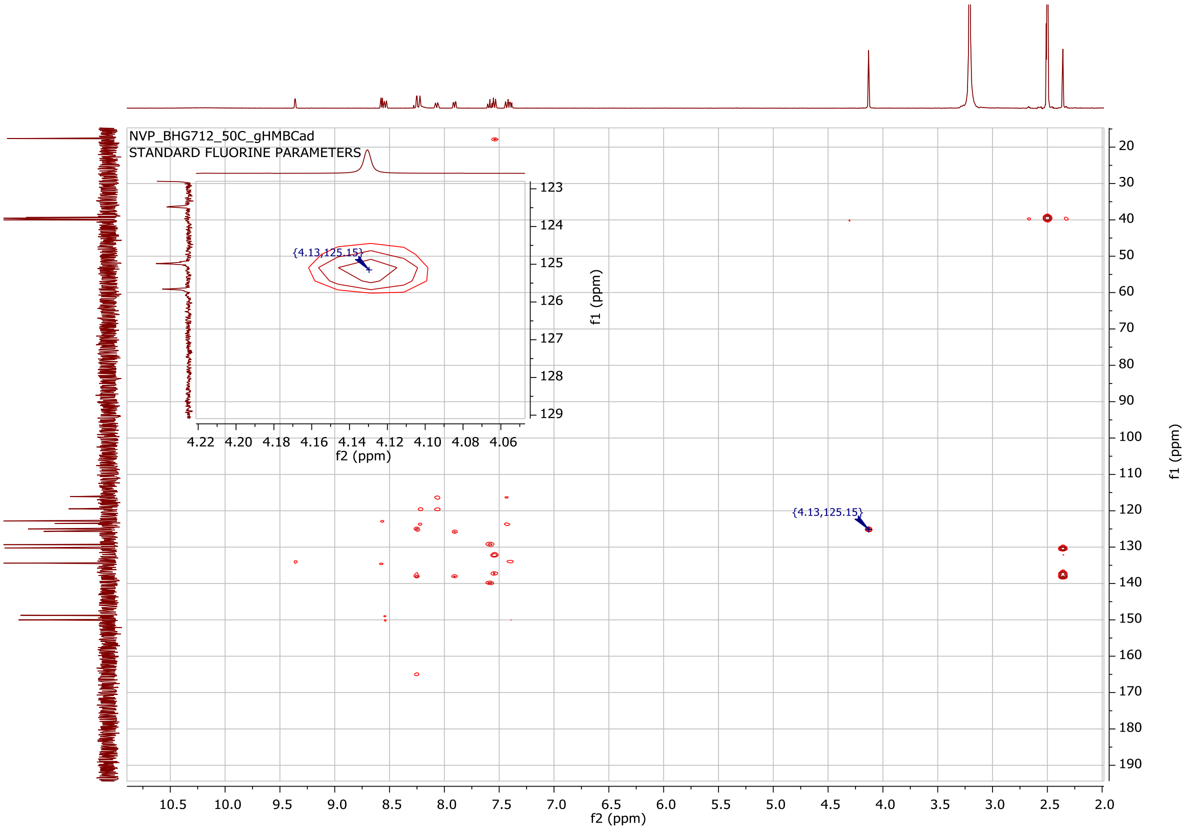

Figure S7. $^{1}H,^{13}C$ HMBC spectrum of commercially acquired 'NVP' at 323 K. The inset highlights the ^{3}J-H,C correlation of the protons attached to the 2-methyl group to the C15 methine carbon in the pyrazole ring at 323 K. The DEPT spectrum is shown on the ^{13}C edge.

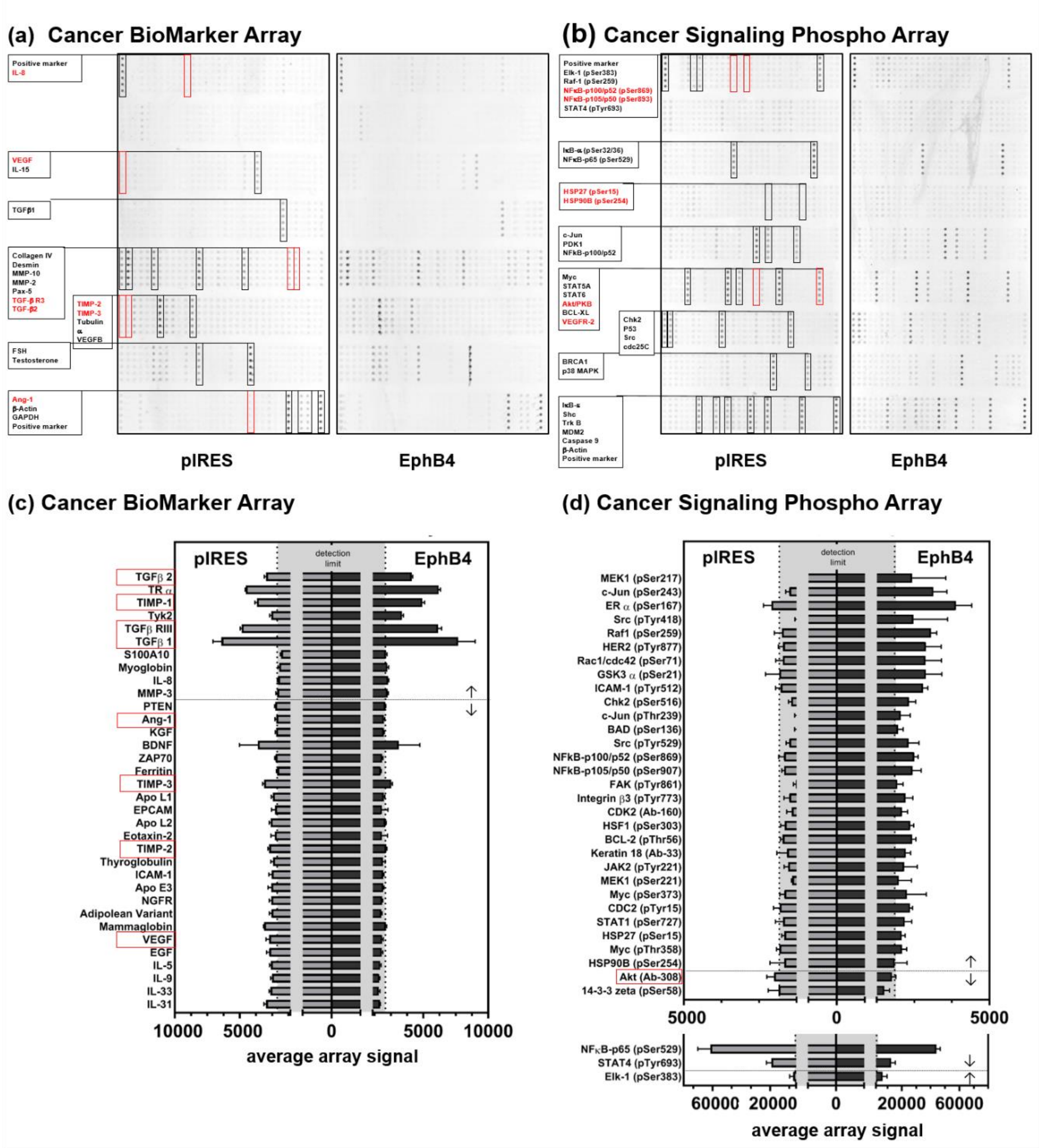

Figure S8. Differential expression and phosphorylation of cancer-related proteins in A375-EphB4 cells in comparison to A375-pIRES cells. (**a/c**) 34 out of 247 analyzed, cancer-relevant proteins differed between A375-pIRES and A375-EphB4 cells including angiogenesis-related proteins (marked in red). (**b/d**) 31 out 136 analyzed phosphorylation variants differed between A375-pIRES and A375-EphB4 cells including angiogenesis-related proteins. (**c/d**) Detection level of the respective array was marked in grey since often one sample (A375-pIRES or A375-EphB4) was beneath detection level. Bars represent means of average array signals from 6 independent spots + SD for A375-pIRES (left panel) and A375-EphB4 cells (right panel).

References

1. Tröster, A.; Heinzlmeir, S.; Berger, B. T.; Gande, S. L.; Saxena, K.; Sreeramulu, S.; Linhard, V.; Nasiri, A. H.; Bolte, M.; Muller, S.; Kuster, B.; Medard, G.; Kudlinzki, D.; Schwalbe, H., NVP-BHG712: Effects of Regioisomers on the Affinity and Selectivity toward the EPHrin Family. *ChemMedChem* **2018,** 13, (16), 1629-1633.
